# Localization of the Center of the Intramuscular Nerve Dense Region of the Suboccipital Muscles: An Anatomical Study

**DOI:** 10.3389/fneur.2022.863446

**Published:** 2022-04-06

**Authors:** Jie Wang, Yanrong Li, Meng Wang, Shengbo Yang

**Affiliations:** ^1^Department of Pain, Affiliated Hospital of Zunyi Medical University, Zunyi, China; ^2^Department of Radiology, Affiliated Hospital of Zunyi Medical University, Zunyi, China; ^3^Department of Anatomy, Zunyi Medical University, Zunyi, China

**Keywords:** suboccipital muscle, center of intramuscular nerve dense region, botulinum toxin A, tension-type headaches, target localization

## Abstract

**Purpose:**

This study aimed to determine the body surface puncture position and depth of the center of the intramuscular nerve dense region in the suboccipital muscle to provide morphological guidance for accurate botulinum toxin A injection to treat headaches caused by increased suboccipital muscle tension.

**Methods:**

Twenty-four cadavers aged 66.5 ± 5.3 years were studied. The curve line connecting occipital eminence or mastoid process and spinous process of the 7th cervical vertebrae was considered the longitudinal reference line (L) and horizontal reference line (H), respectively. Sihler's staining, barium sulfate labeling, and CT were employed. The body surface projection point of the center of the intramuscular nerve dense region was designated as P. The projection of the center of the intramuscular nerve dense region was in the opposite direction across the transverse plane and was recorded as P'. The intersections of the vertical line through point P and lines L and H were designated as P_L_ and P_H_. The percentage position of the P_H_ and P_L_ points on the H and L lines and the depths of the center of intramuscular nerve dense regions were identified.

**Results:**

Sihler's staining showed one intramuscular nerve-dense region in each suboccipital muscle. The P_H_ of the center of the intramuscular nerve dense region was located at 51.40, 45.55, 20.55, and 43.50%. The P_L_ was located at 31.38, 30.08, 16.91, and 52.94%. The depth of the center of the intramuscular nerve dense region was at 22.26, 22.54, 13.14, and 27.30%. These percentage values are all the means.

**Conclusion:**

Accurately defining the body surface position and depth of the center of intramuscular nerve dense region in suboccipital muscles will help to improve botulinum toxin A to target localization efficiency for treating tension-type headache.

## Introduction

Suboccipital muscles coordinate and maintain the posture and stability of the head and neck and bear >50% of the rotation function of the whole head and neck (1, 2). In cases of excessive head rotation or suboccipital muscle spasticity, the vertebral arteries and veins in the suboccipital triangle can be compressed, resulting in headaches due to insufficient intracranial blood supply (3, 4). The anterior fascia of the suboccipital muscle is connected to the dura mater to form a myodural bridge (5, 6). When the suboccipital muscle is chronically strained (7, 8) and the muscle tension increases, a tension-type headache (TTH) can occur (6, 9).

Tension-type headache is the most common primary headache, with a prevalence of 42% (10). However, its pathogenesis remains unclear (11). At present, the interactions between peripheral mechanisms (myofascial nociception) and central mechanisms (inadequate sensitization and endogenous pain control) are considered potential factors in TTH occurrence (12, 13). Continuous tension and strain of neck muscles, especially the suboccipital muscles, cause headaches (14, 15). Persistent muscle tension and contraction aggravate local ischemia and hypoxia, resulting in the release of inflammatory pain-causing substances (16, 17) and exciting and sensitizing peripheral sensory afferents. Muscle spasticity, ischemia, and contractions can also mechanically stimulate nociceptors. Repeated muscle tension and noxious stimulation sensitize the central nervous system, leading to chronic pain (18).

Botulinum toxin A (BTX-A) intramuscular injection to the motor endplate to block the release of acetylcholine from the presynaptic membrane and inhibit muscle excitation has been proven to be an effective method for the treatment of TTH caused by increased suboccipital muscle tension (16, 19–21) and has become increasingly popular (22–24). However, the target location should be accurately identified for curative effects. For small and deep suboccipital muscles, manually locating the block target is impossible. By ultrasound, although the muscle contour can be recognized, the target position (motor endplate or intramuscular nerve branches) cannot be recognized. The electrical stimulator and electromyography indicate the location where the minimum stimulation current leads to the strongest muscle contraction and the area where the electromyography signal is excessively active in the target muscle, respectively (19, 25, 26); however, this requires multiple exploratory punctures, which may be painful for patients. Therefore, obtaining the exact location where BTX-A acts on the suboccipital muscle is crucial. If the motor endplate band is stained, it will be limited by the collection of fresh specimens. However, some reports have revealed that the intramuscular nerve dense region (INDR) was consistent with the motor endplate band's position, which can be used as an alternative target of BTX-A (27–29).

Based on the fact that the location of the INDR and the center of the INDR (CINDR) in suboccipital muscles have not been defined, this study intends to use Sihler's staining to show the INDR, then the CINDR is labeled with barium sulfate, spiral CT scanning and three-dimensional reconstruction are performed to accurately localize the body surface position and depth of CINDR, to provide morphological guidance for the block target localization of BTX-A injection to treat TTH caused by increased tension in the suboccipital muscles.

## Materials and Methods

### Specimens and Ethics

In this study, 24 Chinese adult cadavers (12 men and 12 women) aged 30–75 (66.5 ± 5.3) years at the time of death without a history of neuromuscular diseases and head and neck deformities were collected for analysis. Of the cadavers, 12 were fixed in formalin and 12 were frozen. This study protocol was approved by the Ethics Committee of the Zunyi Medical University (No. #2020-1-006).

### Gross Anatomy Observation and Reference Line Design

In the 12 formalin-fixed cadavers (6 men and 6 women), a transverse incision at the level of the external occipital protuberance and a longitudinal incision from the external occipital protuberance to the spinous process of the 7th cervical spine was made. The skin and subcutaneous tissues were cut as one layer and turned to the front area of the tragus. The trapezius, splenius capitis, and splenius cervicis muscles were exposed by layer, and the fascia was carefully separated to expose the starting and ending points of the suboccipital muscles, the arrangement of muscle fibers, and the source of nerve-muscle branches. We also observed whether there were blood vessels at the location where the nerves enter the muscle. The muscle length, width, and thickness were measured with a Vernier caliper. The external occipital protuberance (point a), the mastoid process (point b), and the spinous process of the 7th cervical spine (point c) were selected as body surface markers. To describe and accurately locate the body surface position and depth of the CINDR of the suboccipital muscles, the curves between points a and c and between points b and c were set as the longitudinal (L) and horizontal (H) reference lines, respectively.

### Modified Sihler's Intramuscular Nerve Staining

The suboccipital muscles of the abovementioned 12 cadavers were completely removed and stained using Sihler's staining process as follows: the muscles were macerated in 3% potassium hydroxide and 0.4% hydrogen peroxide solution for 3–4 weeks; decalcified in Sihler I solution (12 parts of 1% chloral trichlorohydrate, two parts of glacial acetic acid, and two parts of glycerol) for 4 weeks and in Sihler II solution (12 parts of 1% chloral hydrate, two parts of glycerol, and 1 part of Ehrlich's hematoxylin solution) for 4 weeks. It is ideal to decolorize the Sihler I solution for 4–20 h as the muscle was light purple and the nerve branches were black. These were neutralized in 0.05% lithium carbonate solution for 3 h with continuous stirring. For transparency, glycerol gradients of 40, 60, 80, and 100% were performed for 1 week each. The distribution patterns of the intramuscular nerves were observed under an X-ray reading lamp and photographed. The pattern map was drawn and embedded into the corresponding position of the muscle.

### Localization of the CINDR on the Muscle

Using Photoshop cc 2019 (Adobe Photoshop, Adobe Systems Incorporation, California, USA), the dense region of intramuscular nerve branches was framed, the box selection was double-clicked, and the pop-up center point was designated as the CINDR and marked. The percentage position of the CINDR on muscle length (from the nearest origin to the farthest insertion of muscle fiber) and width (from medial to lateral) were measured.

### Spiral CT Localization of the CINDRs

The 12 frozen cadavers (6 men and 6 women) were thawed and dissected to expose the suboccipital muscle. The muscle length and width were measured. Combined with the CINDR position obtained by Sihler's staining, the corresponding position of the CINDR was identified on these suboccipital muscles. The CINDR was marked with barium sulfate (Shandong Jiashuo radiation Protection Engineering Co., Ltd., China), mixed with 801 glue (Wenzhou 801 Glue Corporation Ltd., Wenzhou, China) (according to the proportion of 4 kg/l medical barium sulfate powder and 801 glue) and a layer-by-layer *in-situ* suture was performed. One syringe needle was tied at three bony body surface positions and marked as a, b, and c, respectively, and the barium sulfate-soaked silk threads were sutured on the skin to connect the curve between ac and bc, representing the L and H reference lines, respectively. With the cadaver in the prone neutral position, 64-row spiral CT scanning (Siemens, Germany) (120 kV, 320 mA, collimation of 64 mm × 0.75 mm, and layer thickness of 1 mm) and three-dimensional reconstruction were performed. According to the results of Sihler's staining, one INDR was identified in the rectus capitis posterior minor, rectus capitis posterior major, obliquus capitis superior, and obliquus capitis inferior muscles, respectively. Herein, the CINDRs were named CINDR_1_, CINDR_2_, CINDR_3_, and CINDR_4_, respectively. The CINDR was punctured with a needle perpendicular to the skin and then was rescanned under CT. The projection point of the CINDR on the body surface was named point P, which was the needle puncture point (P_1_, P_2_, P_3_, and P_4_). Using the Syngo system (Siemens, Germany), the length of the H and L reference lines close to the skin was measured. The intersections of the vertical line passing through point P with the H reference line and the horizontal line with the L reference line were recorded as P_H_ (P_1H_, P_2H_, P_3H_, and P_4H_) and P_L_ (P_1L_, P_2L_, P_3L_, and P_4L_). The length between point b and P_H_ was H' (H_1_', H_2_', H_3_', and H_4_'), and the length between point a and P_L_ was L' (L_1_', L_2_', L_3_', and L_4_'). The calculation was H'/H × 100%, L'/L × 100%. On the cross-section, the point on the opposite side skin projected from the vertical skin of point P through the extension line of the CINDR was defined as point P' (P_1_', P_2_', P_3_', and P_4_'). The length of P-CINDR and PP' was measured and calculated by P-CINDR/PP' × 100% and the percentage puncture depth was determined. Then, the located suboccipital muscle was removed and stained with Sihler's staining to verify whether the morphological position of INDR was consistent with that of before.

### Statistical Analysis

The measured data are expressed by the percentage (mean ± SD) of each subject to eliminate the influence of individual differences. The data were processed by SPSS version 18.0 software (SPSS Incorporation, Chicago, Illinois, USA). Based on the normal distribution of the obtained data, the comparison between the left and right sides was tested by a pairing *t-*test, and the sex comparison was tested *via* the *t-*test between two independent samples. The test level was α = 0.05; *P* < 0.05 was considered as statistically significant.

## Results

### Gross Anatomy Observation

**Figure 1:**
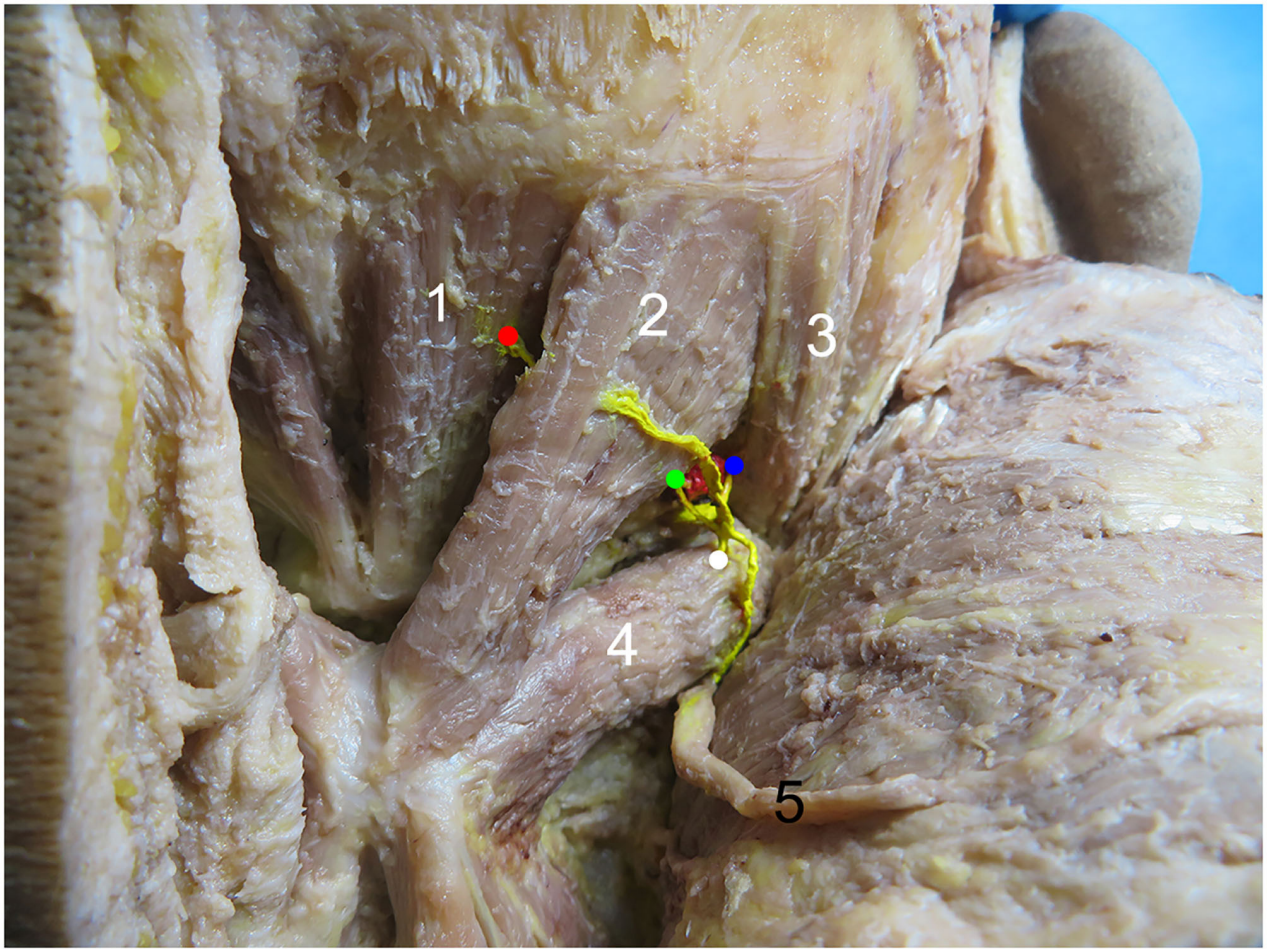
Common types of right suboccipital nerve entry points. 1, Rectus capitis posterior minor muscle. 2, Rectus capitis posterior major muscle. 3, Obliquus capitis superior muscle. 4, Obliquus capitis inferior muscle. 5, Greater occipital nerve. The red, green, blue, and white dots represent the nerve entry points of the rectus capitis posterior minor, rectus capitis posterior major, obliquus capitis superior, and obliquus capitis inferior muscles, respectively.

The rectus capitis posterior major and rectus capitis posterior minor muscles were shaped like inverted triangles, while the obliquus capitis superior and obliquus capitis inferior muscles were shaped like spindles. The rectus capitis posterior minor, rectus capitis posterior major, and obliquus capitis superior muscles all received suboccipital innervation only. Of the obliquus capitis inferior muscles, 95.83% (23/24) were innervated with the suboccipital nerve and the greater occipital nerve, and the branches of the greater occipital nerve were small. Of the rectus capitis posterior minor branches, 83.3% (20/24) first passed through the superficial surface of the rectus capitis posterior major to the center of the abdominal muscle and then entered the muscle on the superolateral surface of the rectus capitis posterior minor. Of the rectus capitis posterior minor branch, 16.7% (4/24) first entered from the head to the outside and below the rectus capitis posterior major, tread on the posterior rectus major of the head, came out of the middle of the rectus capitis posterior major, inclined inward, penetrated the rectus capitis posterior major again, and entered the muscle on the outer and upper surface of the rectus capitis posterior minor muscle. The rectus capitis posterior major branch entered the muscle from the inferolateral side of the deep surface of the muscle. The obliquus capitis superior branch entered the muscle from the lower edge of the muscle. The obliquus capitis inferior branch of the head entered the muscle from approximately 1/3 of the superolateral part of the muscle surface. Blood vessels near each nerve's entry point were observed (Figure 1).

### Distribution Pattern of Intramuscular Nerves in the Suboccipital Muscles

#### The Rectus Capitis Posterior Minor Muscle

**Figure 2:**
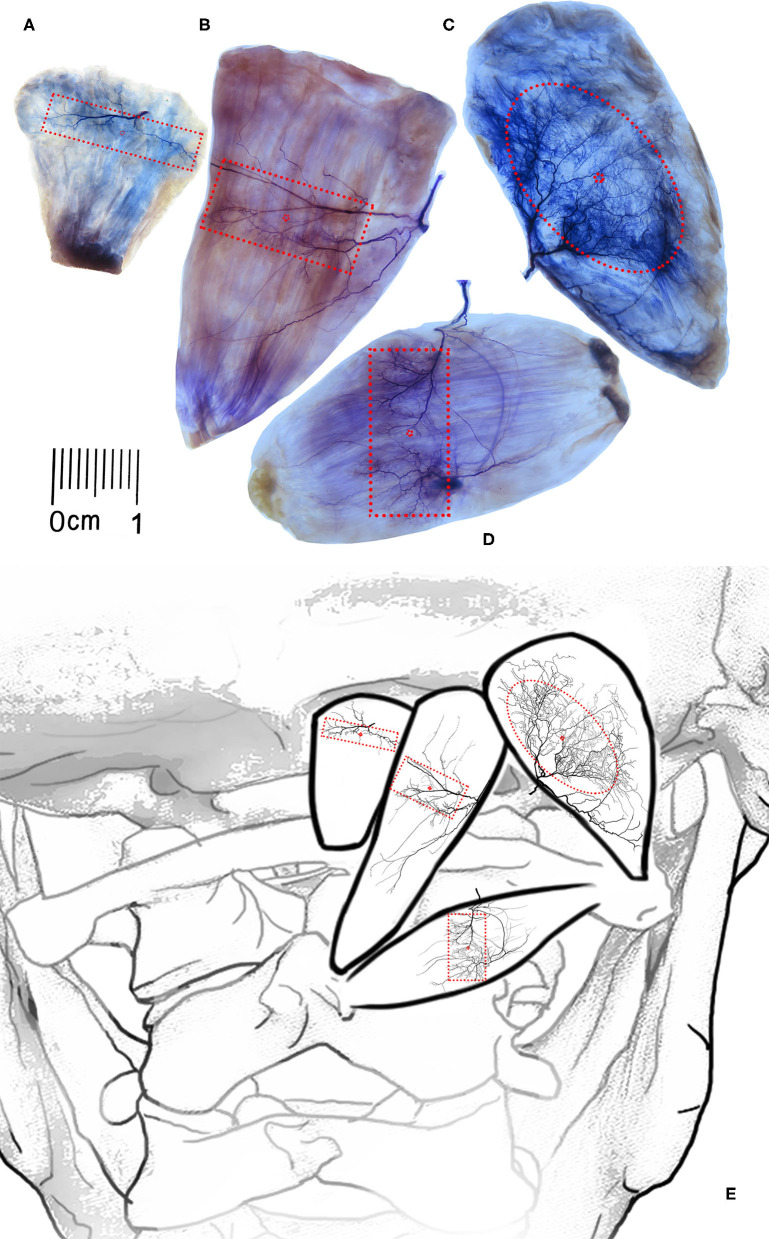
Overall distribution pattern of intramuscular nerves and position of nerve-dense region of the suboccipital muscle. **(A–D)** Showing the Sihler's staining of the rectus capitis posterior minor muscle, rectus capitis posterior major muscle, obliquus capitis superior, and obliquus capitis inferior muscles, respectively. The red boxes showing the intramuscular nerve dense regions, and the red points represent the center of the intramuscular nerve dense regions. Scale: cm. **(E)** A schematic drawing showing the intramuscular position of INDRs and CINDRs.

After the nerve branch of the rectus capitis posterior minor muscle entered the muscle, it divided into two primary branches, which proceeded to the medial edge and lateral edge, respectively. The arborized branches that emerged along the way were densely distributed along the way, forming traffic between branches. At the level of 17.02–46.38% of the muscle length and communicated with each other to form a nerve-dense region of 0.88 ± 0.06 cm^2^, named INDR_1_, and the center of the dense region (CINDR_1_) was located at 31.91 ± 1.18% of the muscle length and 55.41 ± 1.40% of the muscle width (Figures 2A,E).

#### The Rectus Capitis Posterior Major Muscle

The rectus capitis posterior major branch from the dorsal branch of the first cervical nerve was often divided into three primary nerve branches after entering the muscle. One branch crossed the muscle from the superficial surface to the medial border of the muscle and produced small branches along the way to dominate the middle and upper parts of the muscle belly. The second branch advanced toward the middle of the muscle belly and produced many arborized branches at the end, which was anastomosed with the branch of the first primary branch into an INDR, i.e., INDR_2_, at 32.35−49.41% of muscle length, with an area of approximately 1.91 ± 0.12 cm^2^, and the center of the dense area (CINDR_2_) is located at the level of 40.98 ± 1.27% of muscle length and 40.44 ± 1.16% of the muscle width. The third branch progressed toward inferomedial side and the insertion of the rectus capitis posterior major muscle, with relatively few branches (Figures 2B,E).

#### The Obliquus Capitis Superior Muscle

After the obliquus capitis superior muscle branch entered the muscle, it divided into three primary nerve branches, of which one primary nerve branch sent out arborized branches to the upper part of the muscle, and the arborized branches of the second primary branch were distributed in the middle part of the muscle. There was communication between the branches of these two primary branches, forming an oval INDR, i.e., INDR_3_, at 17.76−69.16% of muscle length, with an area of approximately 3.45 ± 0.24 cm^2^, and the center of the dense area (CINDR_3_) was located at 43.93 ± 1.33% of muscle length and 45.83 ± 1.46% of muscle width. The third primary branch progressed toward the inferolateral side and the insertion point of the muscle, with relatively few branches (Figures 2C,E).

#### The Obliquus Capitis Inferior Muscle

After the obliquus capitis inferior muscle nerve branch entered the muscle, it split into three primary nerve branches (medial, middle, and lateral branch). The medial branch was thicker, and its branches were dense in the middle and upper parts of the muscle belly. The middle branch traveled from the deep side of the muscle belly to the middle and lower parts of the muscle belly and began to branch densely. These branches communicated with the arborized branches of the medial branch and formed a rectangular INDR (INDR_4_), which was located at 27.78−59.78% of the muscle length, its area was approximately 1.85 ± 0.13 cm^2^. The center of the nerve-dense region (CINDR_4_) was located at 44.22 ± 2.39% of the muscle length and 48.94 ± 2.11% of the muscle width (Figures 2D,E). The lateral branch was thin, with fewer branches along the way, and its terminal branch communicated with the branches of the middle branch in the lower part of the muscle.

#### Spiral CT Localization of the CINDRs

**Figure 3:**
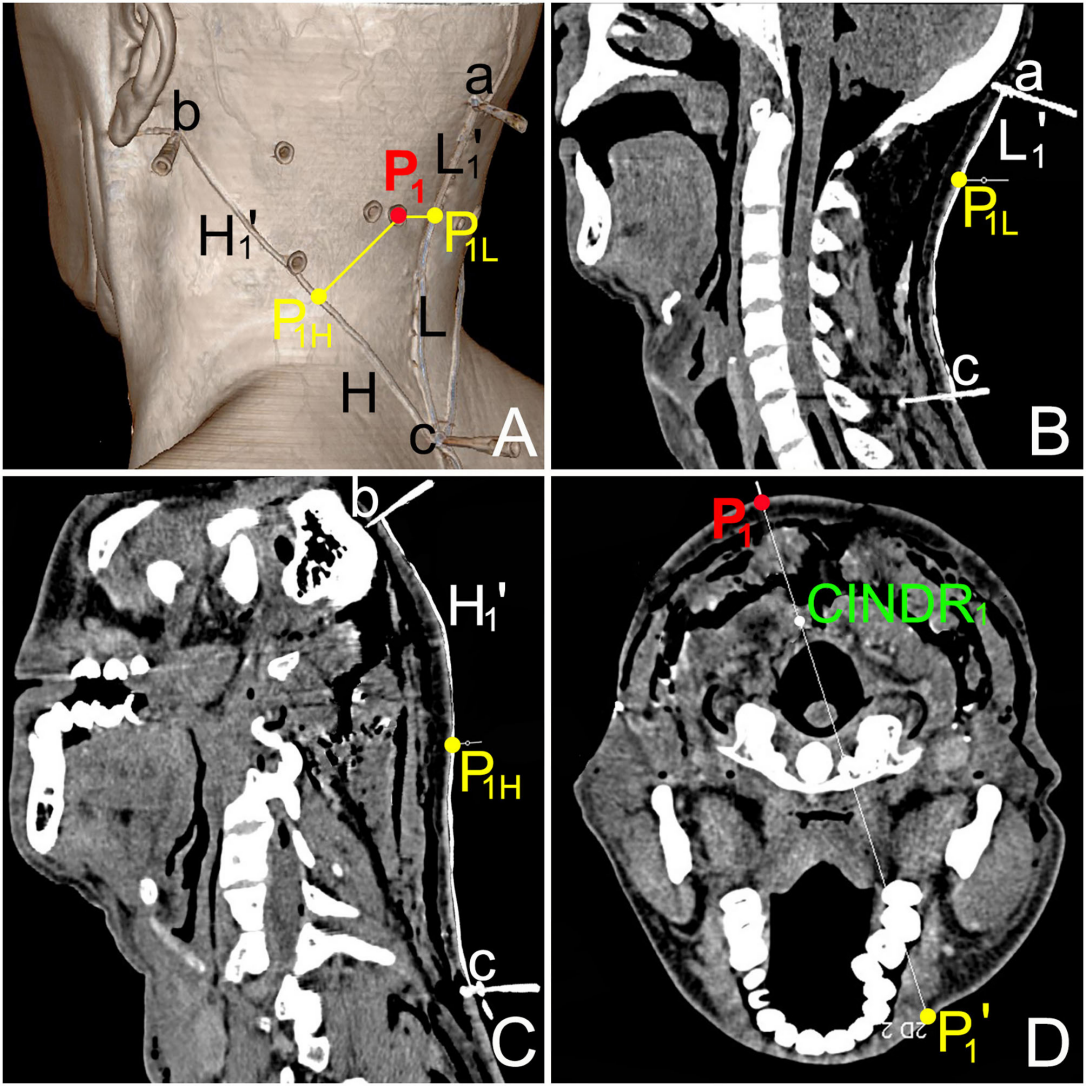
CT images of the center of intramuscular nerve dense region (CINDR_1_) of left rectus capitis posterior minor. **(A)** Spiral CT three-dimensional reconstruction image shows the body surface projection position and design reference line of the CINDR. Point a is the external occipital protuberance, point b is the mastoid process, and point c is the spinous process of the 7th cervical spine. P_1_ is the body surface projection point of CINDR_1_ of rectus capitis posterior minor. ac line = L reference line, a-P_1L_ = L_1_'; bc = H reference line, and b-P_1H_ = H_1_'. **(B)** The length of the L reference line and L_1_' line on the sagittal section through the ac line. **(C)** The length of the H reference line and H_1_' line on the cross-section passing through the bc line. **(D)** The depth of CINDR_1_, measured on the cross-section through P_1_.

The P_H_ of the CINDR of the rectus capitis posterior minor, the rectus capitis posterior major, obliquus capitis superior, and obliquus capitis inferior muscles was located at 51.40 ± 1.56, 45.55 ± 1.23, 20.55 ± 1.42, and 43.50 ± 1.90% of the H reference line, respectively. The P_L_ was located at 31.38 ± 1.15, 30.08 ± 1.34, 16.91 ± 0.72, and 52.94 ± 1.82% of the L reference line, respectively. The depth of the CINDR was located at 22.26 ± 1.18, 22.54 ± 1.51, 13.14 ± 0.72, and 27.30 ± 0.99 of the PP' line, respectively. The absolute depth between CINDR and body surface was 3.78 ± 0.24, 3.56 ± 0.27, 1.71 ± 0.14, and 3.69 ± 0.23 cm, respectively. No statistical difference was observed between the left and right sides and between the sexes (*P* > 0.05) (Tables 1, 2). The CINDR spiral CT localization images of the four suboccipital muscles are illustrated using the rectus capitis posterior minor as an example (Figure 3).

**Table 1:** Comparison of the location of P_L_ and P_H_ of the CINDRs on lines L and H, respectively, and depth of CINDRs between the left and right sides in suboccipital muscles (%).

**CINDR**	**P**_**L**_ **on line L (L'/L)**				**P**_**H**_ **on line H (H'/H)**				**Depth of CINDR (P-CINDR/PP')**			
	**Left (*n =* 12)**	**Right *(n =* 12)**	***t***	***P***	**Left (*n =* 12)**	**Right (*n =* 12)**	***t***	***P***	**Left (*n =* 12)**	**Right (*n =* 12)**	***t***	***P***
CINDR_1_	31.41 ± 1.16	31.04 ± 1.63	0.134	0.896	51.20 ± 1.38	51.59 ± 1.77	−0.783	0.450	22.21 ± 1.25	22.31 ± 1.16	−1.385	0.193
CINDR_2_	30.17 ± 1.42	29.99 ± 1.32	0.367	0.720	45.49 ± 1.32	45.62 ± 1.18	−0.491	0.633	22.40 ± 1.65	22.70 ± 1.42	−1.096	0.296
CINDR_3_	17.01 ± 0.71	16.81 ± 0.75	1.087	0.300	20.40+1.38	20.70 ± 1.50	−0.657	0.525	13.22 ± 0.60	13.05 ± 0.84	0.844	0.417
CINDR_4_	52.84 ± 1.62	53.03 ± 2.06	−0.244	0.812	43.21+1.87	43.80 ± 1.96	−1.340	0.207	27.11 ± 1.13	27.49 ± 0.83	−1.091	0.299

*CINDR, central of the intramuscular nerve region*.

**Table 2:** Comparison of the location of P_L_ and P_H_ of the CINDRs on lines L and H, respectively, and depth of CINDRs between the male and female in suboccipital muscles (%).

**CINDR**	**P**_**L**_ **on line L (L'/L)**				**P**_**H**_ **on line H (H'/H)**				**Depth of CINDR (P-CINDR/PP')**			
	**Male (*n =* 12)**	**Female (*n =* 12)**	***t***	***P***	**Male (*n =* 12)**	**Female (*n =* 12)**	***t***	***P***	**Male (*n =* 12)**	**Female (*n =* 12)**	***t***	***P***
CINDR_1_	30.07 ± 0.87	31.69 ± 1.35	−1.339	0.194	51.24 ± 1.42	51.55 ± 1.74	−0.484	0.633	22.44 ± 1.10	22.08 ± 1.27	0.741	0.467
CINDR_2_	29.68 ± 0.94	30.48 ± 1.59	−1.506	0.146	45.42 ± 1.09	45.68 ± 1.39	−0.515	0.611	22.33 ± 1.28	22.76 ± 1.75	−0.680	0.503
CINDR_3_	16.85 ± 0.75	16.97 ± 0.72	−0.416	0.681	20.73 ± 1.23	20.37 ± 1.61	0.613	0.546	12.99 ± 0.67	13.28 ± 0.76	−1.006	0.325
CINDR_4_	53.19 ± 1.59	52.69 ± 2.06	0.661	0.516	43.54 ± 1.54	43.46 ± 2.27	0.099	0.922	27.08 ± 0.71	27.53 ± 1.19	−1.141	0.266

*CINDR, central of the intramuscular nerve region*.

## Discussion

The tension in the suboccipital muscle can be due to the anatomical relationship of the myodural bridge. When its muscle tension increases, TTH can occur (14, 15, 30). Therapeutic measures can block the release of acetylcholine at the motor endplate by intramuscular injection of BTX-A, reducing muscle tension, and alleviating headaches. Therefore, this study exposed the suboccipital muscle through gross anatomy, Sihler's staining revealed the distribution pattern of intramuscular nerves using barium sulfate-labeling, spiral CT scanning, and three-dimensional reconstruction and accurately localized the body surface position and puncture depth of intramuscular nerve block targets of these muscles. It provides an important morphological basis for BTX-A injection in the treatment of TTH caused by increased tension of the suboccipital muscle.

In addition to headaches caused by the suboccipital muscles, abnormal cervical muscle tension headaches are also associated with splenius capitis and splenius cervicis muscles. The intramuscular nerve distribution pattern of these two muscles has been revealed by Sihler's staining. The distribution of the nerve in the middle part of the muscle belly of the splenius capitis was the densest, indicating that 50% of the muscle length should be the most effective area for the administration of the BTX-A injection. The intramuscular nerve of the splenius cervicis muscle was widely distributed between 30 and 70% of the muscle length (31). Although this study describes the location of the INDRs in the muscle, it did not locate the medial-lateral relationship and superior-inferior relationship between CINDR and body surface landmarks and puncture depth.

This experiment's results indicate that the INDR of the obliquus capitis inferior muscle is close to the middle part muscle belly and the INDR of the other three suboccipital muscles was not located in the middle part of the muscle belly. Nerve endings and muscle fibers often form motor endplates in the middle of the muscle fibers, and spindle muscles composed of isometric muscle fibers often form the motor endplate band in the middle part of the muscle belly. Therefore, the middle of the muscle belly is often the target of BTX-A injections (32, 33). However, for different muscles, the shape and position of the motor endplate band were different. As shown in the result, the INDR of most muscles was not in the middle part of the muscle belly. Therefore, revealing the accurate location of the motor endplate band or INDR is crucial to improving the efficacy of the BTX-A injection. Studies have revealed that the efficacy of BTX-A depends on the distance between the injection target and the motor endplate. If the position of the target deviates from the motor endplate by 5 mm, the efficacy will reduce by 50% (34). Moreover, when BTX-A is injected beyond the effective target, the toxin will spread to the surrounding muscle, resulting in nonspasmodic muscle paralysis (28, 35).

When BTX-A is injected clinically to treat patients with TTH, 3–4 sites are often selected for injection at the local tenderness point (pain point) of the suboccipital muscle and the dose at each site is 15–20 U. However, the specific injection site and dose of each suboccipital muscle have not been described in detail (36, 37). Studies have reported that 1 U of BTX-A may diffuse to 1.5–3 cm^2^ at the site of injection, whereas 2.5–5 U can diffuse to 4.5 cm^2^ at the site of injection (27, 38). Combined with the area calculation of INDR in this study, it can be calculated that the required dose of BTX-A is only 1–1.5 U for the obliquus capitis inferior muscle, 2–3 U for the obliquus capitis superior, 1–1.5 U for the rectus capitis posterior major, and 0.5–1 U for the rectus capitis posterior minor muscle. This suggests that as long as the target location is accurate, the drug dose will be greatly reduced, which can reduce not only the costs for patients but also the risk of drug infiltration into the surrounding tissues.

In conclusion, Sihler's staining was used to demonstrate the intramuscular nerve distribution pattern of the suboccipital muscle. Using barium sulfate to label the center of the INDR, spiral CT scanning, and designed longitudinal and transverse reference lines, the geometric relationship between bone landmarks and the CINDR was established. The CINDR was projected onto the body surface, and its puncture position and depth data are expressed as a percentage, which can eliminate individual differences to make the clinical operation strong. Thus, the results of this study will help to improve the target localization efficiency of BTX-A in the treatment of headache caused by increased tension of the suboccipital muscle.

However, this study has some limitations that should be noted. The ethnic differences of the patients were not considered and the curative effect needs to be verified by clinical application. In addition, this study was conducted in normal subjects, and not in TTH ex-sufferers. We believe that ultrasound can be used to assist in clinical localization to improve the efficiency and curative effect of the target block. Injection of deep suboccipital muscles without ultrasound guidance may be accompanied by an increased risk of injuring the vertebral artery especially if this artery is elongated in the individual.

## Conclusion

This study is the first study to systematically investigate the intramuscular nerve dense region and to define the body surface position and depth of center of the intramuscular nerve dense region in suboccipital muscles. It will help improve BTX-A target localization efficiency for treating tension-type headache.

## Data Availability Statement

The original contributions presented in the study are included in the article/supplementary material, further inquiries can be directed to the corresponding author/s.

## Ethics Statement

The studies involving human participants were reviewed and approved by the Ethics Committee of Zunyi Medical University (No. #2020-1-006). The patients/participants provided their written informed consent to participate in this study.

## Author Contributions

SY conceived and designed the experiments, performed the experiments, contributed to writing, reviewing, and editing the manuscript. JW conceived and designed the experiments, performed the experiments, analyzed the data, and wrote the original draft. YL and MW contributed to reagents, materials, and analysis tools. All authors contributed to the article and approved the submitted version.

## Funding

This study was supported by the National Natural Science Foundation of China (No. 31660294), the Guizhou Science and Technology Project (No. ZK [2021] 115), and the Guizhou Provincial Health Commission (No. gzwkj2021-277).

## Conflict of Interest

The authors declare that the research was conducted in the absence of any commercial or financial relationships that could be construed as a potential conflict of interest.

## Publisher's Note

All claims expressed in this article are solely those of the authors and do not necessarily represent those of their affiliated organizations, or those of the publisher, the editors and the reviewers. Any product that may be evaluated in this article, or claim that may be made by its manufacturer, is not guaranteed or endorsed by the publisher.
